# Acute liver failure associated with human adenovirus infection after allogeneic hematopoietic stem cell transplantation

**DOI:** 10.1007/s00277-023-05253-y

**Published:** 2023-05-13

**Authors:** Zhimin Lin, Yanjun Wu, Ye Zhao, Tingjing Wang, Jing Xia, Huiying Qiu, Zhengming Jin, Depei Wu, Feng Chen

**Affiliations:** 1 National Clinical Research Center for Hematologic Diseases, Jiangsu Institute of Hematology, The First Affiliated Hospital of Soochow University, Suzhou, China; 2 Institute of Blood and Marrow Transplantation, Collaborative Innovation Center of Hematology, Soochow University, Suzhou, China; 3 Key Laboratory of Stem Cells and Biomedical Materials of Jiangsu Province and Chinese Ministry of Science and Technology, Suzhou, China

Dear Editor,

The incidence of HAdV infection in the post-transplantation population is approximately 3–47%, and the incidence after allo-HSCT (5–47%) is much higher than that after auto-HSCT (2.5–14%) [1, 2]. The hepatitis is mainly associated with HAdV subtype C, especially serotype C 5 [3]. We would like to report two cases of hepatitis caused by HAdV infection after allo-HSCT in our hospital, which rapidly progressed to acute liver failure.

The patient 1, a male, 30 years old, was diagnosed as “early T precursor lymphocytic leukemia (ETP-ALL)” a second time in February 2020 with a history of “peripheral T-cell lymphoma Stage IV.” After preconditioning with the TBI-Cy regimen, a haploidentical donor hematopoietic stem cell transplantation was performed in a state of NR on June 4 and 5, 2020. Plasma CMV-DNA positive (+ 28 days, + 40 days, + 88 days) and EBV-DNA positive (+ 133 days, + 146 days) were diagnosed as CMVemia and EBVemia, all of which turned negative after antiviral therapy. On December 22, 2020 (more than six months after allo-HSCT), the patient developed a fever without obvious inducement, accompanied by loss of appetite. Liver function tests were normal, with no abnormalities in blood culture, routine tests, and imaging examinations. Empiric anti-infective therapy and low-dose dexamethasone were administered. On December 28, the patient still had a fever, fatigue, mild abdominal pain, and diarrhea. Laboratory indicators suggested liver dysfunction for the first time (Table 1). The routine screening of the hepatitis virus group showed negative results for HAV, HBV, HCV, HDV, and HEV, and the PB mNGS was tested on December 31. Suspected liver injury drugs were discontinued, and liver-protecting drugs was given. The patient gradually developed abdominal distension, advanced fatigue, nasal bleeding, and oliguria. With active supportive treatment, blood transfusion, IVIg, and albumin infusion; however, the patient’s condition deteriorated. On January 2, 2021, he developed irritability, and complained of chest tightness, accompanied by nausea and vomiting. Physical examination showed hemorrhagic spots in the skin mucosa, mild edema in the face and legs, and abdominal distention, without flapping tremors. An urgent blood ammonia test showed 286 μmol/L. The patient suffered respiratory and cardiac arrest, failed to rescue, and died in the evening. PB mNGS suggested 71,112 reads of HAdV group C, including 14,312 reads of C5 and 111 reads of C6. The final clinical diagnosis was acute severe human adenovirus hepatitis, and acute liver failure.

**Table 1:** Part of the liver function of the patient 1

	12–28 (d1)	12–30 (d3)	12–31 (d4)	01–01 (d5)	01–02 (d6)
T-BIL	6.8	7.4	7.1	15.5	30.8
ALT	92	201	380	740	2097
AST	108	319	610	1675	7358
LDH	604	716	918	1682	4958
γ-GGT	260	371	559	686	699

The patient 2, a 33-year-old male, was diagnosed as “acute myeloid leukemia” in November 2021. After preconditioning with the modified BUCY regimen, an unrelated donor (male to male, O + to O +) hematopoietic stem cell transplantation was performed in the state of CR on April 15, 2022. At + 14 days, skin aGVHD (grade II) appeared and improved after methylprednisolone treatment. At + 45 days, EBVemia was diagnosed and turned negative after antiviral therapy. On July 21, 2022, Azacitidine was treated in combination with Venetolcax. On July 30, 2022 (+ 106 days after allo-HSCT), he developed a fever with a maximum temperature of 37.8 °C, nausea, and vomiting occurred the next day. The biochemical test suggested liver dysfunction: ALT 97 U/L, AST 117 U/L, and LDH 329 U/L. Empiric anti-infection and hepatoprotective drugs were given. The patient still had recurrent fever with nausea and vomiting. Liver enzymes increased further, but the bacterial culture and qt-PCR tests of CMV and EBV DNA were negative. Anti-infective drugs were adjusted, and then, the body temperature was controlled after the addition of small doses of methylprednisolone, but the liver enzymes continued to increase. On August 12 (+ 118 days),he developed upper abdominal pain accompanied by nausea and vomiting. CT showed multiple small patches of slightly low-density shadows in the liver, which were newly found. Upper abdominal MRI showed hepatic hemochromatosis and multiple nodular abnormal signal foci in hepatic parenchyma. Two days later, nausea and vomiting continued, and even had a drop in blood pressure (66/41 mmHg). Ecchymosis was seen on the abdomen. He was treated with dopamine, blood transfusion, and plasma exchange. Fever recurred on August 15, with the highest temperature of 38.6 °C, dizziness, and pain all over the body. The next day, condition deteriorated again, including coma, hypotension, and sighed-like breathing. He died after giving up treatment. PB mNGS suggested 86,615 reads of HAdV group C. Finally, he was clinically diagnosed as acute severe human adenovirus hepatitis, and acute liver failure (Table 2).

**Table 2:** Part of the liver function of the patient 2

	8–8 (d1)	8–9 (d2)	8–10 (d3)	8–11 (d4)	8–12 (d5)	8–13 (d6)	8–14 (d7)	8–15 (d8)
ALT	211	289	400	464	473	464	471	494
AST	266	348	473	599	831	599	648	8110
LDH	376	428	488	555	647	555	2809	10745
γ-GGT	108	127	167	207	261	207	347	139
T-BIL	8.2	7.7	8.2	8.9	9.4	8.9	35.6	42.7

Annotations: T-BIL (μmol/L); ALT (U/L); AST (U/L); LDH (U/L); γ-GGT (U/L)

The two patients developed the disease in early stage after transplantation and progressed rapidly. They both were treated with ATG before transplantation, and both were treated with a myeloablative preconditioning regimen. They were characterized by fever, fatigue, and poor appetite, and the results of auxiliary examinations mainly showed coagulopathy and liver enzymes increased significantly. These are generally consistent with previous reports [4]. The liver enzymes increased significantly, mainly the AST, which may be a feature of HAdV infection. HAdV group C5 has a good affinity for hepatocytes. It has shown that coagulation factors containing GLA domain can mediate the entry of the virus into hepatocytes by binding to major viral capsid proteins, such as FX, FIX, and FVI, among which FX has the highest affinity with the capsid protein of the HAdV group C5 [5, 6]. This may be part of the reason for the severe coagulopathy. The diagnosis of HAdV hepatitis, which we reported, was analyzed by mNGS technology. A case of post-transplant adenovirus infection–associated acute liver failure diagnosed by mNGS was also reported at Shanghai People’s Hospital [7]. The mNGS technology is the second generation of metagenomic sequencing technology. Our previous studies have shown that mNGS is more effective than traditional pathogen detection methods in patients with suspected infection after HSCT[8]. It is especially suitable for the screening of patients with critical illness, rare pathogen infections, and immune deficiency. Even if HAdV infection is diagnosed at a late stage, there is no time for specific antiviral therapy and it does not respond to conventional hepatoprotective medication. Routine ADV DNA screening can be performed in high-risk populations by qPCR technology, as well as EBV DNA and CMV DNA monitoring. Common anti-HAdV drugs include Cidofovir (CDV) and Brincidofovir (BDV). Adoptive T cell therapy, an alternative therapy in which virus-specific memory T cells (VST) are injected into the recipient to destroy the virus, is effective at low doses [9]. Also, the emerging T cell receptor-engineered T cell therapy (TCR-T) can alter donor T cell specificity at the gene level. By introducing the CRISPR-Cas9 gene into initial human cells, protective LTDL-specific TCR is successfully inserted into endogenous TCR sites while effectively eliminating endogenous TCR, achieving the goal of changing the specificity of T cells [10]. There are also HAdV-related vaccines in development, but HAdV vaccines contain activated HAdV 4 subtype and 7 subtype, which limits their use in post-transplant patients [11].

Our cases are of particular interest because HAdV hepatitis after allo-HSCT is relatively rare, and they can provide references for early diagnosis of the disease, which may be helpful to further improve the survival rate of patients after allo-HSCT.

## Data Availability

The datasets during and analyzed during the current study are available from the corresponding author on reasonable request.
